# A snapshot of plasma metabolites in first-episode schizophrenia: a capillary electrophoresis time-of-flight mass spectrometry study

**DOI:** 10.1038/tp.2014.19

**Published:** 2014-04-08

**Authors:** S Koike, M Bundo, K Iwamoto, M Suga, H Kuwabara, Y Ohashi, K Shinoda, Y Takano, N Iwashiro, Y Satomura, T Nagai, T Natsubori, M Tada, H Yamasue, K Kasai

**Affiliations:** 1 Department of Neuropsychiatry, Graduate School of Medicine, The University of Tokyo, Tokyo, Japan; 2 Division for Counseling and Support, The University of Tokyo, Tokyo, Japan; 3 Department of Molecular Psychiatry, Graduate School of Medicine, The University of Tokyo, Tokyo, Japan; 4 Department of Child Neuropsychiatry, Graduate School of Medicine, The University of Tokyo, Tokyo, Japan; 5 Human Metabolome Technologies, Tsuruoka, Japan; 6 Japan Science and Technology Agency, CREST, Tokyo, Japan; 7 Japan Science and Technology Agency, National Bioscience Database Center (NBDC), Tokyo, Japan

## Abstract

Few biomarkers have been known that can easily measure clinical conditions in mental illnesses such as schizophrenia. Capillary electrophoresis time-of-flight mass spectrometry (CE-TOFMS) is a new method that can measure ionized and low-molecular-weight metabolites. To explore global metabolomic alterations that characterize the onset of schizophrenia and identify biomarkers, we profiled the relative and absolute concentrations of the plasma metabolites from 30 patients with first-episode schizophrenia (FESZ, four drug-naïve samples), 38 healthy controls and 15 individuals with autism spectrum disorders using CE-TOFMS. Five metabolites had robust changes (increased creatine and decreased betaine, nonanoic acid, benzoic acid and perillic acid) in two independent sample sets. Altered levels of these metabolites are consistent with well-known hypotheses regarding abnormalities of the homocysteine metabolism, creatine kinase-emia and oxidative stress. Although it should be considered that most patients with FESZ received medication, these metabolites are candidate biomarkers to improve the determination of diagnosis, severity and clinical stages, especially for FESZ.

## Introduction

Schizophrenia is a syndrome characterized by positive, negative symptoms and cognitive dysfunction with enduring social deficits, for which patients' needs for objective indices and effective treatments have not been fully met. Approximately 0.7% of the general population is affected by schizophrenia and disease onset is most common during adolescence or early adulthood. The World Health Organization reported that the estimated burden of schizophrenia accounts for 2.3% of all diseases over the world, and disability-adjusted life year is ranked ninth in all non-communicable diseases.^1^ A recent survey from the general public in diverse communities showed that acute schizophrenia was the heaviest burden in all diseases.^2^ Numerous studies aimed at elucidating the pathogenesis of schizophrenia have hypothesized involvement of abnormalities of the central nervous system, such as the dopaminergic and glutamatergic systems^3^ and/or abnormalities in metabolic systems, such as the glycolytic system, oxidative stress and excessive immune response(s).^4, 5, 6, 7, 8^

Shortening duration of untreated psychosis (DUP), a lag from the onset of psychotic symptoms to receiving effective treatment, would provide better symptomatic and functional outcomes.^9,10^ However, early detection for schizophrenia is hard when psychiatrists determine only using clinical investigations, because clinical manifestations before the onset of schizophrenia consist of mostly nonspecific symptoms like depression and anxiety. In addition, more specific symptoms like positive and disorganized symptoms in the early clinical stages are partially shared with autism spectrum disorders (ASD).^11, 12, 13^ Promising biomarkers are needed to improve the determination of diagnosis, severity and clinical stage, as well as for the prediction of symptomatic and functional outcomes.^14,15^

Metabolomics measures the concentration of metabolites in a given sample, such as serum and cerebrospinal fluid. Because metabolite status reflects the pathophysiological status of a subject, the metabolomics approach can be used to identify candidate molecule(s) for biomarkers of psychiatric disorders, especially before and after the onset of clinical manifestations. However, measurable metabolites and the resolution of those measurements are largely method-dependent. In patients with schizophrenia, nuclear magnetic resonance,^4,5,16^ liquid chromatography mass spectrometry (MS)^4,8,17^ and gas chromatography MS^5,7^ have been used to analyze metabolites in samples including postmortem brain specimens, cerebrospinal fluid and serum and plasma derived from peripheral blood. These studies have attempted to elucidate the pathogeneses of schizophrenia and have explored possible biomarkers for diagnosis.

Capillary electrophoresis time-of-flight MS (CE-TOFMS) is a recently developed, state-of-the-art, metabolome analysis tool.^18^ The major advantages of CE-TOFMS analysis include extremely high resolution, versatility (that is, ability to analyze metabolic profiles of various organisms), and ability to simultaneously quantify virtually all the charged low-molecular-weight compounds in a sample.^19,20^ The efficacy of CE-TOFMS has been demonstrated in various human clinical studies.^21,22^ As metabolome analysis using CE-TOFMS has not been conducted in schizophrenia, it is expected that CE-TOMS can identify novel candidate metabolites, with potential to be used as biomarkers for the disease. Therefore, the purpose of this study was to identify metabolites whose plasma levels were altered in patients with first-episode schizophrenia (FESZ) compared with control subjects, using CE-TOFMS. Demonstrating altered levels of metabolites in patients with FESZ would lead to indicate the molecular signature at the onset of psychosis, and to identify potential biomarkers for the determination of diagnosis, severity and clinical stages, as well as the prediction of outcomes.

## Materials and methods

### Study participants

A total of 83 Japanese individuals (all Asian ethnicity) participated in this study. Two independent sets of samples were enrolled for exploration and replication. The first set was sampled from March 2009 to November 2010 and the second set from December 2010 to July 2012. Eighteen patients and 14 age- and sex-matched control subjects were assigned to the first set; whereas 12 patients, 24 age- and sex-matched controls and 15 age-matched individuals with drug-naïve ASD were included in the second set (Table 1 and Supplementary Table s1). Two patients with FESZ in the first set and two in the second set served as drug-naïve participants. In addition, one patient in the first set was naïve to antipsychotics at the time of blood sampling. This study is a part of the IN-STEP (Integrative Neuroimaging studies in Schizophrenia Targeting for Early Intervention and Prevention) project.^23^ The participants were recruited from the outpatient and inpatient units of The University of Tokyo Hospital, University of Tokyo Health Service Center, psychiatry clinics and internet referrals. The control participants were recruited from internet referral, message board in several universities and voluntary recruitment in The University of Tokyo Hospital. All eligible participants were diagnosed using the DSM-IV-TR (Diagnostic and Statistical Manual of Mental Disorders, Fourth Edition, Text Revision).^24^ The inclusion criteria were age 15–40 years, no use of antipsychotic medications (for the purpose of controlling schizophrenia) for >32 cumulative weeks (median 8 weeks, range 0–32 weeks), and presence of continuous psychotic symptoms for the past 60 months.^25^ The exclusion criteria were presence or history of neurological illness, traumatic brain injury with any known cognitive consequences or loss of consciousness for >5 min, a history of electroconvulsive therapy, low premorbid IQ (<70), previous history of alcohol addiction, previous continuous illegal substance use (for example, cannabis) and clearly diagnosed as ASD and dissociative disorders. For the control group, additional exclusion criteria included any current or previous history of psychiatric disease detected by screening with the modified Mini-International Neuropsychiatric Interview^26^ or a family history of any axis I disorder(s) within a first-degree relative. All patients were evaluated using the GAF (Global Assessment of Functioning)^27^ scale and the Positive and Negative Syndrome Scale (PANSS)^28^ scores on the day of the experiment. If the patients were being treated with antipsychotics, benzodiazepines and/or anti-Parkinsonian agents, doses were calculated as equivalent doses of chlorpromazine, diazepam and biperiden, respectively. For the exploration of disease specific differences, plasma samples were also obtained from age-matched, drug-naïve, individuals with ASD from the second set. Exclusion criteria for the ASD group were the same as that used for the FESZ group, with the exception that participants with a full-scale IQ<80, on the basis of the revised form of the WAIS-R (Wechsler Adult Intelligence Scale) or WAIS-III, were excluded. The inclusion and exclusion criteria, diagnostic protocols and clinical assessments of participants with ASD were the same as those used in a previous study.^12,29,30^ Briefly, after >2 months of follow-up examinations, an experienced psychiatrist (HY) carefully diagnosed the participants with ASD strictly on the basis of the DSM-IV-TR criteria. A different child adolescent psychiatrist (HK) confirmed the diagnoses using the Japanese version of the Autism Diagnostic Interview-Revised.^31^ Data regarding the ASD group has been reported elsewhere,^12^ and were used in this study for discriminant analysis. All participants provided written informed consent after being given a complete explanation of the study, as required by the ethics committee of The University of Tokyo Hospital (No. 2094-[6] and 2226-[2]).

### CE-TOFMS analysis

Peripheral blood samples were drawn by experienced physicians, from a peripheral vein while the patient was fasting (>3 h without any meal and/or nutritious drink). Within 30 min from blood collection, plasma samples were isolated via centrifugation at 1200 *g* for 10 min, and then stored at −80 °C until use. The first set of samples was analyzed from December 2010 to May 2011 and the second set was analyzed from August 2012 to October 2012.

Plasma samples (100 μl) were plunged into 0.45 ml methanol containing 10 μM each methionine sulfone and 10-camphorsulfonic acid and mixed well. Then, 200 μl deionized water and 0.5 ml chloroform were added and the solution was centrifuged at 2300 g for 5 min at 4 °C. The upper aqueous layer was centrifugally filtered through a 5-kDa cutoff filter (Human Metabolome Technologies, Tsuruoka, Japan) to remove proteins. The filtrate was lyophilized and dissolved in 50 μl of ultrapure water containing reference compounds before MS analysis. The water was produced by a Milli-Q Academic A10 (EMD Millipore, Billerica, MA, USA).

Samples were applied to a capillary electrophoresis system equipped with an Agilent 6210 Time-of-flight mass spectrometer (CE-TOFMS, Agilent Technologies, Santa Clara, CA, USA), as previously described.^32^ Raw data files from CE-TOFMS were processed using custom, proprietary software written in Java (an extended version of MathDAMP which has been developed in Keio University).^33^ The software performs (1) peak picking and (2) peak alignment. For (1), all peaks potentially corresponding to metabolites are extracted. After peak picking, the migration time of electrophoresis was normalized using those of the internal standards. For (2), an alignment was applied according to similar mass-to-charge ratios (*m/z*) and normalized migration times. The tolerance was set to 100 p.p.m. (*m/z*) and 0.5 min (normalized migration time). The peak matrix was matched with the annotation table of the metabolomic library (Human Metabolome Technologies) described previously on the basis of their *m/z* and migration times.^34^

### Statistical analysis

For each metabolite, the relative area was defined as the relative concentration of that metabolite. As CE-TOFMS can identify a small amount of relative areas of metabolites, several metabolites were not detected across the samples. As these data could not be distinguished whether due to subthreshold value or zero value of this system, one half of the minimum measure for that sample was imputed.^35^ In the first set, concentrations were compared between patients with FESZ and control subjects using a two-tailed Mann–Whitney *U*-test. In the second set, for the purpose of replicating the significant differences identified in the first set, the relative concentrations of these metabolites were compared between patients with FESZ and control subjects using a one-tailed Mann–Whitney *U*-test. Step-wise discriminant analysis, using the metabolites that showed significant differences in the first set, was used to identify the metabolites capable of discriminating patients from control subjects. Discriminant analysis was then performed on data from the second set using the concentrations of the metabolites identified in the first set. In the second set, discriminant analysis between individuals with FESZ and ASD was also performed to confirm specificity of the identified metabolites for individual diseases. In patients with FESZ, correlation coefficients between clinical symptoms and significant metabolites were calculated using two-tailed (for the first set) and one-tailed (for the second set) Spearman's rank correlation coefficients. For DUP, logarithmic transformation, to correct for skewed data, was conducted and the transformed data were used for further analysis.^9^ CE-TOFMS analysis using the current system enables measurement of the absolute quantities of 108 pre-determined major metabolites in each sample on the basis of the peak area of internal controls of each metabolite. The quantity of these metabolites can be reliably compared across different experimental batches. Therefore, the two-tailed *U*-test and Spearman's rank correlation coefficients were used to test the absolute concentrations of the major metabolites between the FESZ and control groups, by combination of the first and second sets. The threshold for statistical significance was set to *P*<0.05 for all analyses. Statistical analyses were conducted using SPSS, version 20.0 (IBM, Armonk, NY, USA).

## Results

### Demographic characteristics

Demographic characteristics in the first set and second set were shown in Table 1 and Supplementary Table s1. Twenty-four patients with FESZ were diagnosed as schizophrenia, two were delusional disorder and four were psychotic disorder, not otherwise specified. Clinical outcomes at 6 months were obtained for 11/18 patients with FESZ in the first set. Wilcoxon-Matched-Pairs tests showed that all clinical variables at 6 months were improved from baseline (6-month PANSS positive: 10.9±4.1, *P*=0.011; PANSS negative: 13.5±5.3, *P*=0.029; PANSS general pathology: 23.9±6.5, *P*=0.0044; GAF: 50.0±13.7, *P*=0.0093).

### Group differences

**Figure 1:**
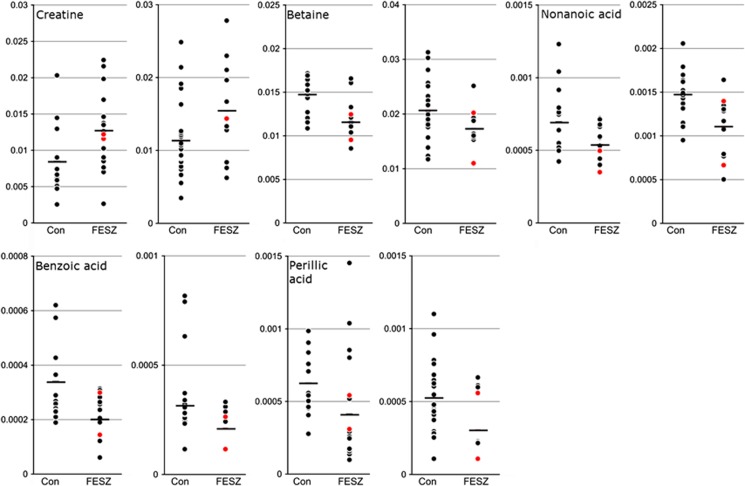
Relative concentrations of analytes that showed significant differences. Y axis indicates relative concentrations. Bars indicate mean concentration in the group and red plots indicate the drug-naïve patients in the first (left) and second (right) sets. Con, controls; FESZ, patients with first-episode schizophrenia.

Full data sets were shown in Supplementary Table s1. A total of 175 metabolites were detected in the plasma of the first set (112 and 63 metabolites for cationic and anion modes, respectively). Nine metabolites showed significantly different relative areas between patients with FESZ and control subjects (*P*<0.05, Table 2). In the second set, 142 metabolites were detected (76 and 66 metabolites for cationic and anion modes, respectively). Of the nine metabolites identified in the first set, patients with FESZ had a significant increase in creatine (*P*=0.031) and significant decreases in betaine, nonanoic acid, benzoic acid and perillic acid (*P*=0.029, 0.00031, 0.039, 0.0040, respectively) in the second set (Figure 1,Table 2). All changes detected in the second set were in the same direction as the changes identified in the first set. Of the remaining four metabolites identified in the first set, two metabolites showed similar trends in patients with FESZ in the second set (glutamate, *P*=0.064 and gluconic acid, *P*=0.052) and two (imidazoleacetic acid and cyclohexylamine) were not detected in either diagnostic group in the second set.

Of the nine significant altered metabolites listed in the first set, four metabolites (creatine, glutamate, gluconic acid, and betaine) were included as pre-determined major metabolites in the CE-TOFMS analysis procedure. Therefore, the absolute concentrations of these four metabolites were available and compared using a combined data set including 30 patients with FESZ and 38 control subjects. The concentrations of creatine, gluconic acid and betaine were significantly different between the FESZ and control groups (*P*=0.011, 0.0043 and 0.00013, respectively, Table 3) and the concentration of glutamate showed a similar trend (*P*=0.25, Table 3).

### Discriminant analysis

In the first set, step-wise discriminant analysis between the FESZ and control groups showed that the diagnosis of 87.5% (28/32) of the subjects could be correctly classified using three metabolites (betaine, glutamate and nonanoic acid; Wilks' *λ*=0.417, *P*<0.001). Validation performed using data from the second set showed that the diagnosis of 83.3% (30/36) of the subjects could be correctly classified (Wilks' *λ*=0.566, *P*<0.001). This result was applied to data from subjects with ASD and showed that the diagnosis of 81.5% (22/27) of the subjects with ASD could be discriminated from patients with FESZ.

Correlation analysis showed that creatine and glutamate could be affected by medications (assessed below). Therefore, the same discriminant analyses were conducted without creatine and glutamate. Step-wise discriminant analysis in the first set showed that the diagnosis of 81.2% (26/32) of the subjects could be correctly classified using three metabolites (betaine, gluconic acid and nonanoic acid; Wilks' *λ*=0.449, *P*<0.001). In the second set, the diagnosis of 77.8% (24/36) of the subjects was correctly classified (Wilks' *λ*=0.530, *P*<0.001) and that of 81.5% (22/27) of the individuals with ASD could be discriminated from patients with FESZ.

### Relationship between metabolite concentration and clinical assessment

**Figure 2:**
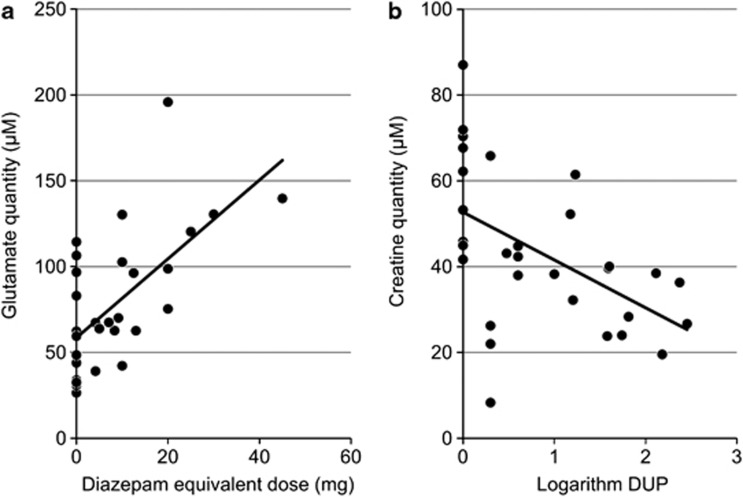
Correlation coefficients between absolute concentration and clinical variables. (**a**) Correlation between glutamate concentration and diazepam equivalent dose (*ρ*=0.613, *P*=0.0003). (**b**) Correlation between creatine concentration and logarithm DUP (*ρ*=−0.617, *P*=0.0003). DUP, duration of untreated schizophrenia (week).

The correlational analysis using Spearman's rank correlation coefficients in the first (two-tailed), second (one-tailed) and combination sets (two-tailed) showed significant correlations between plasma glutamate and the diazepam equivalent dose (first: *ρ*=0.688, *P*=0.0016; second: *ρ*=0.657, *P*=0.010; combination: *ρ*=0.613, *P*=0.00032, Figure 2a) and between plasma creatine and logDUP (first: *ρ*=−0.603, *P*=0.0077; second: *ρ*=−0.606, *P*=0.018; combination: *ρ*=−0.617, *P*=0.00028, Figure 2b). Linear regression analysis using the combination set showed that creatine concentration was separately predicted by chlorpromazine dose and logDUP (chlorpromazine: *β*=0.35 (95% confidence interval (CI)=0.001–0.74), *P*=0.049; logDUP: *β*=−0.44 (95% CI=−0.78–0.10), *P*=0.012). In the first set, reduced betaine was associated with severe positive symptoms (*ρ*=−0.527, *P*=0.024), but this correlation was not replicated in the second set nor combination set.

Preliminary analysis showed that the creatine concentration was correlated with the PANSS general psychopathology subscore (*ρ*=0.763, *P*=0.0063) and with the PANSS total score (*ρ*=0.624, *P*=0.040). In addition, concentrations of nonanoic acid and perillic acid were correlated with the PANSS general psychopathology subscore (*ρ*=0.671, *P*=0.024 and *ρ*=0.639, *P*=0.034, respectively).

### Results of medication effects on metabolites

We divided the patients with schizophrenia into two subgroups whether patients used antipsychotics or not, and also made subgroups in accordance with usage of benzodiazepines and anti-parkinsonian, respectively. In the first set, glutamate concentration in the patients using benzodiazepines (*n*=8) was significantly lower than that in the patients using no benzodiazepine (*n*=10, *U*=12.0, *P*=0.012). In the second set, any significant change was not shown between patients using antipsychotics, benzodiazepines, anti-Parkinsonian drugs or not. In absolute concentration analysis of four metabolites, glutamate concentration in the patients using benzodiazepines (*n*=17) was significantly lower than that in the patients using no benzodiazepine (*n*=13, *U*=51.0, *P*=0.012). Glutamate concentration in the patients using anti-parkinsonian (*n*=13) was also significantly lower than that in the patients using no anti-parkinsonian (*n*=17, *U*=58.0, *P*=0.028).

## Discussion

Metabolome analysis using CE-TOFMS revealed that the plasma concentrations of several metabolites were different in patients with FESZ compared with control subjects. Although most patients with FESZ received medication and should be carefully considered, two independent sample sets were analyzed and increased levels of creatine, and decreased levels of betaine, nonanoic acid, benzoic acid and perillic acid were identified in patients with FESZ. By quantifying the absolute concentration of major metabolites, the changes in the concentrations of three metabolites (creatine, gluconic acid and betaine) were validated in patients with FESZ. To the best of our knowledge, this is the first study that elucidates possible biomarkers for the onset of schizophrenia, using peripheral blood plasma and the CE-TOFMS system.

Betaine (trimethylglycine) serves as an osmoregulator and is a substrate in the betaine-homocysteine methyltransferase reaction, which converts homocysteine to methionine.^36^ In human tissues, this reaction is essential for homocysteine metabolism and methyl-donor transportation pathways, such as DNA methylation.^36, 37, 38^ Excess homocysteine in the brain leads to elevated oxidative stress and inflammatory cytokine level, and eventually to neural toxicity and microvascular damage.^39,40^ Congenital homocystinuria is similarly associated with systemic illnesses such as thrombosis, osteoporosis, mental retardation and sometimes, psychotic symptoms.^41,42^ Betaine supplements have been used in the treatment of congenital homocystinuria and have also been shown effective in reduction of hyperhomocysteinemia in healthy individuals.^37,43^ The C677T polymorphism in the methylenetetrahydrofolate reductase gene is a risk factor for hyperhomocysteinemia^44,45^ and is associated with psychiatric diseases such as bipolar disorders,^38^ depression^38,46^ and schizophrenia.^38,46,47^ In this study, patients with FESZ demonstrated a significantly decreased betaine concentration in both data sets (first set and second set). Interestingly, in the first set, reduced betaine was associated with severe positive symptoms. Although this correlation was not replicated in the second set, this may be attributable to significantly lower PANSS positive scores in the second set. Although future studies are needed to discover the reason(s) underlying the decreased betaine concentration in patients with FESZ, decreased methylenetetrahydrofolate reductase function and/or aberrant homocysteine synthesis may result in the downregulation of a betaine-related pathway in schizophrenia.

Nonanoic acid is a nine-carbon, chained, monocarboxylic acid, and occurs naturally as an ester in the oil made from *pelargonium*. Although little is known regarding the action of nonanoic acid in humans, nonanoic acid is a family of medium-chain fatty acids including valproic acid, which is used to treat manic symptoms in patients with bipolar disorder and schizophrenia. One possible common role for mood stabilizers, including valproic acid, is a reduction in the inositol phosphate level through inhibition of PI3K (phosphatidylinositol 3-kinase).^48,49^ Interestingly, nonanoic acid inhibits PI3K activity more than valproic acid.^48^ The finding that patients with FESZ have reduced concentrations of nonanoic acid suggests that abnormal inositol phosphate signaling may have a role in schizophrenia.

Glutamate is an amino acid and is well known as the primary excitatory neurotransmitter in the brain. Numerous studies have hypothesized altered activity of the glutamatergic system in schizophrenia.^50, 51, 52^ However, glutamate levels measured in peripheral blood samples from patients with schizophrenia have been inconsistent across studies, in that some studies have reported increased levels,^5,7,53^ nonsignificant changes^51,54^ or decreased levels.^4,55,56^ Longitudinal studies have shown that blood glutamate levels increase during the clinical course of antipsychotics medication and clinical improvement.^51,54,56^ Results from the current study showed trends toward increased plasma glutamate levels in patients with FESZ. In addition, current results showed an association between the level of glutamate and benzodiazepine equivalent doses. Because medication doses were significantly associated with the severity of symptoms at onset, and there was no difference in glutamate concentration with benzodiazepine treatment in the second set, it is difficult to conclude that medications directly affect the glutamate concentration in peripheral blood.^50^ More studies are necessary to fully elucidate the relationship between medication and glutamate concentrations.

Previous studies have shown that a marked spike in the serum creatine kinase level, the so-called PACK (schizophrenia-associated creatine kinase-emia), occurs especially at the onset of schizophrenia.^57, 58, 59^ PACK was obscured in chronic stages of schizophrenia, suggesting that the leak of creatine kinase from muscle to blood occurred especially around the onset of schizophrenia.^57,58^ The current results showed that the creatine concentration in patients with FESZ increased compared with controls and was negatively associated with DUP. These results suggest that patients showed altered creatine metabolism only proximate to the onset of schizophrenia. Furthermore, preliminary results showed that an increased creatine concentration was associated with severe symptoms at 6 months. As DUP was one of the most promising outcome indicators for schizophrenia, the plasma creatine concentration and its changes in schizophrenia may be a potential biomarker used to detect clinical stage and to predict symptomatic outcome.

It should be noted that this study had several limitations. As only a small number of drug-naïve patients with FESZ participated in this study, treatment effects and metabolism changes due to medication could not fully be eliminated. As several metabolites were significantly correlated with medication doses, further longitudinal studies, with drug-naïve patients, will be needed. However, consistent differences could not be detected when Mann–Whitney *U-*tests were used to compare patients using antipsychotics, benzodiazepines or anti-parkinsonian agents and patients not using medications. In addition, successful classification was achieved using only metabolites whose levels were not apparently affected by medication. Second, although the current results could be useful for discrimination of subjects with FESZ and ASD, other psychiatric disorders (for example, depression and bipolar disorder) have to be considered due to the similar pathophysiology of these psychiatric disorders. Third, in addition to medication effect, potential confounding of dietary food before sample collection should be considered. Fourth, longitudinal measurements of metabolites with detailed clinical investigations will be needed to clarify if the identified metabolites show state- or trait-dependent changes. Finally, although one of the major advantages of CE-TOFMS analysis is able to simultaneously quantify all the charged low-molecular-weight compounds in a sample, we have to measure all the metabolites in a pathway combining other MS such as liquid chromatography MS^4,8,17^ and gas chromatography MS.^5,7^

In conclusion, small and ionic metabolites were measured in plasma from peripheral blood using the CE-TOFMS system. Patients with FESZ had increased levels of creatine, and decreased levels of betaine, nonanoic acid, benzoic acid and perillic acid. Although most patients with FESZ received medication, altered levels of these metabolites were specific in patients with FESZ, and consistent with well-known hypotheses regarding abnormalities of the homocysteine metabolism, PACK and oxidative stress. These metabolites could become biomarkers used for differential diagnosis, determination of clinical stage, prediction of outcomes and treatment responses. Furthermore, these biomarkers could promote earlier detection and better treatment options, eventually leading to a better prognosis.

## Figures and Tables

**Table 1:** Demographic characteristics in this study

	*First set*			*Second set*					
	*FESZ mean (s.d.)*	*Controls*	P-*value*^a^	*FESZ*	*Controls*	*ASD*	P*-value*a,^b^	P-*value*a,^c^	P-*value*a,^d^
Participants, n (male)	18 (13)	14 (11)	0.50	12	24 (10)	15 (15)	0.28	0.001^†^	0.094
Age (years)	23.2 (5.4)	25.7 (6.1)	0.17	24.6 (7.1)	26.1 (2.6)	28.6 (5.3)	0.49	0.10	0.63
Smoking, %^e^	11.1	7.1	1.0	25.0	5.0	NA	0.14	NA	0.32
Age at onset (years)	22.1 (5.4)	NA	NA	24.1 (7.2)	NA	NA	NA	NA	0.44
DUP (weeks)	47.3 (84.9)	NA	NA	22.5 (43.3)	NA	NA	NA	NA	0.55
Duration of illness (weeks)	11.2 (9.7)	NA	NA	13.9 (15.6)	NA	NA	NA	NA	1.0
GAF	35.6 (11.6)	NA	NA	43.8 (17.4)	NA	NA	NA	NA	0.25
PANSS positive	17.1 (4.2)	NA	NA	12.8 (5.4)	NA	NA	NA	NA	0.028*
PANSS negative	22.1 (8.2)	NA	NA	17.6 (8.5)	NA	NA	NA	NA	0.11
PANSS general pathology	36.8 (9.0)	NA	NA	32.6 (10.2)	NA	NA	NA	NA	0.23
Chlorepromazine dose (mg)	497 (440)	NA	NA	609 (634)	NA	0 (0)	NA	0.007^†^	0.92
Diazepam dose (mg)	6.6 (8.1)	NA	NA	11.2 (14.2)	NA	0.0 (0.0)	NA	0.019*	0.52
Biperiden dose (mg)	1.5 (2.5)	NA	NA	1.7 (2.2)	NA	0.0 (0.0)	NA	0.023*	0.47

Abbreviations: ASD, autism spectrum disorders; DUP, duration of untreated psychosis; FESZ, first-episode schizophrenia; GAF, the global assessment of functioning; NA, not applicable; PANSS, the positive and negative symptom scale. *P<0.05, ^†^*P*<0.01.

a Mann–Whitney *U*-test or Fisher's exact test.

b FESZ versus controls.

c FESZ versus ASD. As we used the data of ASD only for comparing with those of FESZ, we did not test using analysis of variance.

d First set versus second set in the patients with FESZ.

e We could not obtain the data of smoking in four control participants in the second set.

**Table 2:** A list of metabolites that showed significant difference between the FESZ and control groups

*Metabolites*	*Mode*	*m/z, p.p.m.*	*MT, min*	*First set*			*Second set*				
				*Mean relative area (s.d.)*		P-*value*^a^	*Mean relative area (s.d.)*			P-*value*	
				*FESZ (*n*=18)*	*Con (*n*=14)*		*FESZ (*n*=12)*	*Con (*n*=24)*	*ASD (*n*=15)*	*FESZ vs Con*^b^	*FESZ vs ASDb,^c^*
Creatine^d^	C	132.08	9.000	0.01270 (0.00512)	0.00845 (0.00493)	0.018*	0.0154 (0.0065)	0.0114 (0.0052)	0.0118 (0.0057)	0.031*	0.052
Gluconic acid^d^	A	195.05	7.809	0.000325 (0.000060)	0.000280 (0.000044)	0.022*	0.000256 (0.000049)	0.000228 (0.000038)	0.000205 (0.000043)	0.052	0.0060†
Glutamate^d^	C	148.06	11.090	0.01007 (0.00044)	0.00698 (0.00239)	0.049*	0.0210 (0.0078)	0.0156 (0.0109)	0.0164 (0.0065)	0.064	0.057
Benzoic acid	A	121.03	9.482	0.000201 (0.000093)	0.000340 (0.000157)	0.037*	0.000211 (0.000087)	0.000315 (0.000183)	0.000387 (0.000259)	0.039*	0.034*
Imidazolelactic acid	C	157.06	8.978	0.0000554 (0.0000247)	0.0000746 (0.0000300)	0.037*	ND	ND	ND	NA	NA
Nonanoic acid	A	157.12	7.774	0.000538 (0.000121)	0.000695 (0.000235)	0.025*	0.00110 (0.00034)	0.00147 (0.00023)	0.00144 (0.00020)	0.00031†	0.0031†
Perillic acid	A	165.09	7.808	0.000413 (0.000386)	0.000626 (0.000211)	0.018*	0.000303 (0.000232)	0.000526 (0.000234)	0.000325 (0.000232)	0.0040†	0.20
Cyclohexylamine	C	100.11	7.837	0.0000528 (0.0000608)	0.0000918 (0.0000816)	0.0018^†^	0.000031 (0.000008)	ND	ND	NA	NA
Betaine	C	118.09	11.407	0.0116 (0.0021)	0.0147 (0.0021)	0.00068^†^	0.0173 (0.0040)	0.0206 (0.0051)	0.0220 (0.0039)	0.029*	0.0013†

Abbreviations: A, anion mode; ASD, autism spectrum disorders; C, cation mode; Con, control; FESZ, first-episode schizophrenia; MT, migration time (min); m/z, mass-to-charge ratio (p.p.m.); NA, not applicable; ND, not detected.

*P<0.05, ^†^*P*<0.01.

a Two-tailed Mann–Whitney *U*-test.

b One-tailed Mann–Whitney *U*-test.

c As we used the data of ASD only for comparing with those of FESZ, we did not test using analysis of variance.

d Increased metabolites in patients with FESZ compared with the controls in the first set.

**Table 3:** Absolute quantities of the metabolites that showed significant difference between the FESZ and control groups

*Metabolites*	*Mean (s.d.), μM*		P*-value^a^*
	*FESZ (*n*=30)*	*Con (*n*=38)*	
Creatine	43.2 (18.10)	32.22 (16.42)	0.006^†^
Gluconic acid	3.732 (0.687)	3.295 (0.534)	0.006^†^
Glutamate	77.93 (39.86)	65.19 (48.71)	0.057
Betaine	43.00 (12.34)	56.99 (15.35)	0.00013^†^

Abbreviations: Con, control; FESZ, first-episode schizophrenia.

*P<0.05, ^†^*P*<0.01.

a Two-tailed Mann–Whitney *U*-test.

## Notes

YO and KS have been employed by Human Metabolome Technologies that undertook CE-TOFMS analysis in this study. The remaining authors declare no conflict of interest.
