# Supplementary material for: A snapshot of plasma metabolites in first-episode schizophrenia: a capillary electrophoresis time-of-flight mass spectrometry study
Source: Transl Psychiatry. 2014 Apr 8;4(4):e379–. doi: 10.1038/tp.2014.19 (PMC4012283; doi:10.1038/tp.2014.19)
Supplement: Supplementary Table [file tp201419x1.doc]

Table s1. The number of diagnosis in each set.

|  | First Set |  | Second Set |
| --- | --- | --- | --- |
| 295.1 Schizophrenia, disorganized type | 4 |  | 4 |
| 295.3 Schizophrenia, paranoid type | 7 |  | 3 |
| 295.4 Schizophreniform disorder | 3 |  | 3 |
| 297.1 Delusional disorder | 2 |  | 0 |
| 298.9 Psychotic disorder not otherwise specified | 2 |  | 2 |
